# Bioactivities of Lipid Extracts and Complex Lipids from Seaweeds: Current Knowledge and Future Prospects

**DOI:** 10.3390/md19120686

**Published:** 2021-11-30

**Authors:** Diana Lopes, Felisa Rey, Miguel C. Leal, Ana I. Lillebø, Ricardo Calado, Maria Rosário Domingues

**Affiliations:** 1 Centre for Environmental and Marine Studies, CESAM, Department of Chemistry, Santiago University Campus, University of Aveiro, 3810-193 Aveiro, Portugal; dianasalzedaslopes@ua.pt (D.L.); felisa.rey@ua.pt (F.R.); 2 Mass Spectrometry Centre, LAQV-REQUIMTE, Department of Chemistry, Santiago University Campus, University of Aveiro, 3810-193 Aveiro, Portugal; 3 ECOMARE, Centre for Environmental and Marine Studies, CESAM, Department of Biology, Santiago University Campus, University of Aveiro, 3810-193 Aveiro, Portugal; miguelcleal@ua.pt (M.C.L.); lillebo@ua.pt (A.I.L.); rjcalado@ua.pt (R.C.)

**Keywords:** algae, bioactivity, glycolipids, lipidomics, macroalgae, phospholipids, seaweeds

## Abstract

While complex lipids of seaweeds are known to display important phytochemical properties, their full potential is yet to be explored. This review summarizes the findings of a systematic survey of scientific publications spanning over the years 2000 to January 2021 retrieved from Web of Science (WoS) and Scopus databases to map the state of the art and identify knowledge gaps on the relationship between the complex lipids of seaweeds and their reported bioactivities. Eligible publications (270 in total) were classified in five categories according to the type of studies using seaweeds as raw biomass (category 1); studies using organic extracts (category 2); studies using organic extracts with identified complex lipids (category 3); studies of extracts enriched in isolated groups or classes of complex lipids (category 4); and studies of isolated complex lipids molecular species (category 5), organized by seaweed phyla and reported bioactivities. Studies that identified the molecular composition of these bioactive compounds in detail (29 in total) were selected and described according to their bioactivities (antitumor, anti-inflammatory, antimicrobial, and others). Overall, to date, the value for seaweeds in terms of health and wellness effects were found to be mostly based on empirical knowledge. Although lipids from seaweeds are little explored, the published work showed the potential of lipid extracts, fractions, and complex lipids from seaweeds as functional ingredients for the food and feed, cosmeceutical, and pharmaceutical industries. This knowledge will boost the use of the chemical diversity of seaweeds for innovative value-added products and new biotechnological applications.

## 1. Introduction

Marine macroalgae, popularly known as seaweeds, have emerged as one of the contributors to achieve United Nations sustainable development goals (SDG) [1]. Indeed, algae can be used in healthy and sustainable diets, thereby meeting the farm to fork strategy, which is the core of the European Green Deal [2,3]. Moreover, they are a rich source of nutrients and valuable bioactive phytochemicals that act as preventive agents against non-communicable diseases [4] and that can contribute to overcome multiple societal challenges, such as the ongoing fight on obesity [5] and on the issues caused by antimicrobial resistance in microorganisms [6,7]. Additionally, their chemical diversity can also be paramount to fight infectious viral diseases and allow a higher efficiency when tackling future pandemic situations [7,8]. The exploitation of seaweeds as marine resources for new high value-added products thus contributes to increase their economic relevance on multiple niche markets [9].

Seaweeds, have been used since earliest times as a source of food and in traditional medicine in Asian and other seacoast countries around the world [1]. Although their generalized value for human nutrition and health is already recognized, it is mostly based on empirical knowledge. Seaweeds are reservoirs of bioactive compounds [10] yet to be fully used in a plethora of blue biotechnology applications [11], such as functional foods and feeds, pharmaceutical, nutraceutical [12], cosmeceutical [13], and other high-end uses.

Well-known phytochemicals have already been recorded from seaweeds, including polysaccharides, proteins, pigments, and other minor compounds such as phenolics and vitamins [14]. Seaweed lipids are a less abundant fraction of such bioactive phytochemicals that, despite their great value, remain largely over-looked, likely because of their lower content, high structural diversity, and complexity, along with a rather poorly understood biological activity. They are mainly known as reservoirs of omega-3 polyunsaturated fatty acids (PUFA) with well-recognized health benefits [15]. Nevertheless, seaweeds also have complex lipids, such as phospholipids (PLs) and glycolipids (GLs), which display unique features that are not found in terrestrial plants, such as being esterified with omega-3 fatty acids (FA), including eicosapentaenoic acid (EPA) and docosahexaenoic acid (DHA) [16,17]. Marine PLs have better bioavailability, resistance to oxidation, and higher content of omega-3 PUFA than lipids from other sources. Moreover, they are better at delivering dietary omega-3 PUFA than terrestrial PLs, as already demonstrated in several comparative studies [18,19,20,21]. On the other hand, and unlike their terrestrial analogues, GLs from seaweeds contain long chain PUFA (20 or more carbon atoms) with potential biotechnological applications [22]. PLs and GLs play a structural role in biological systems, representing the major building blocks of cytoplasmatic and chloroplast membranes [23]. They are also the main carriers of PUFA [24,25].

**Figure 1:**
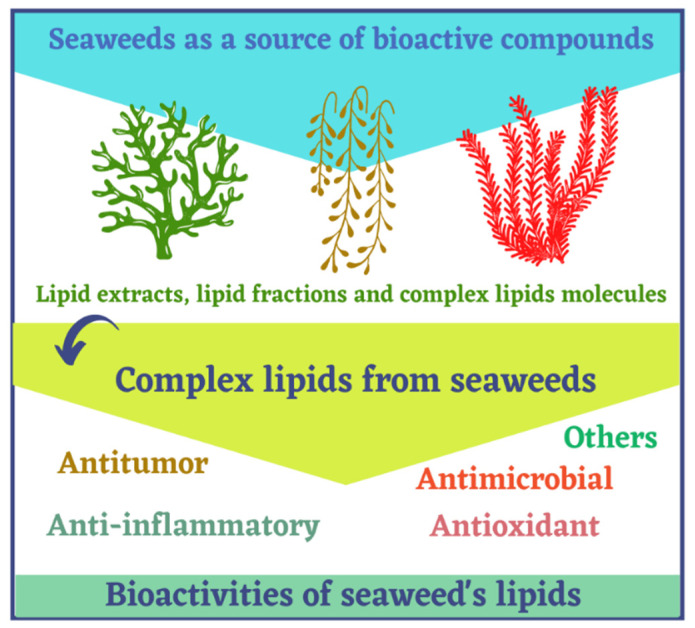
Complex lipids from seaweeds as bioactive compounds with reported bioactivities.

Recently, complex lipids are being considered, promising phytochemicals with intrinsic bioactive properties, including antioxidant, antitumor, anti-inflammatory, and antimicrobial [7,26,27], fostering potential applications in pharmaceutical, nutraceutical, and cosmeceutical fields (Figure 1) [28]. However, the complexity and structural diversity of complex lipids are hindering their detailed characterization and exploitation. Most published works describing seaweed bioactive lipids refer to assays of total lipid extracts or enriched fractions [29,30,31], and few studies are focused on the identification and characterization of complex lipids, making it difficult to establish a clear structure–activity relationship. Nevertheless, the rapid development of modern -omics approaches and bioinformatic tools in recent years have been contributing to achieve a detailed mapping of the lipidome of seaweeds from different phyla. Selected species to date include *Ulva rigida* and *Codium tomentosum* from Chlorophyta phylum [32,33]; *Chondrus crispus*, *Palmaria palmata*, *Porphyra dioica*, *Gracilaria* sp. from Rhodophyta phylum [27,34,35,36]; and *Fucus vesiculosus*, *Saccharina latissima*, *Sargassum muticum*, and *Bifurcaria bifurcata* from Ochrophyta phylum [37,38,39]. The comparison of seaweeds lipidome revealed unique lipid signatures [40]. While some phylum-specific trends could perceived, lipidomic signatures were rather species-specific [40]. More work is needed to achieve a larger coverage of seaweeds lipidome to fully unravel the specificity of their signatures and support value added uses of these marine bioresources.

Despite its biotechnological potential, our knowledge on naturally occurring bioactive complex lipids from seaweeds is still in its infancy. Only recently sustainably farmed seaweeds have emerged in Europe [41]. The production of seaweeds biomass under controlled conditions has promoted the safeguarding of high food safety standards, and subsequently generated interest in the bioprospecting for new compounds, namely complex lipids, for high-end biotechnological uses. For now, questions such as the relationship between bioactivities already detected and complex lipid structures and their specificity remain to be answered.

The authors have performed a systematic review of scientific literature to establish the state of the art of our knowledge on naturally occurring bioactive complex lipids from seaweeds. The information here assembled provides new insights on how studies are being performed and allows the identification of gaps in knowledge that still need attention in upcoming years.

## 2. Methods

**Figure 2:**
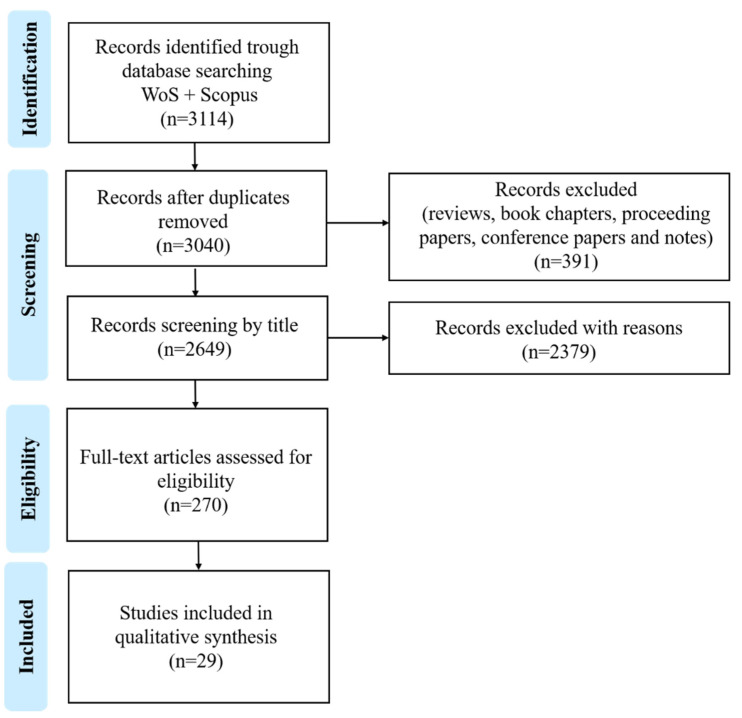
Schematic review selection process performed according to PRISMA 2020 flow diagram [42].

This systematic review followed the Preferred Reporting Items for Systematic Reviews and Meta-Analysis (PRISMA-P) guidelines [42]. We used two databases to retrieve scientific publications: Web of Science (WoS) (www.webofknowledge.com, accessed on 21 January 2021) and Scopus (www.scopus.com, accessed on 25 January 2021). A comprehensive search on the bioactivity of complex lipids from seaweeds was performed based on a query by topic (title, abstract and keywords) of the terms: ((alga* OR seaweed* OR macroalga*) AND (“complex lipid*” OR lipid* OR glycolipid* OR phospholipid*) AND (bioactiv* OR activ*)); spanning over the years 2000 to January 2021. The search query resulted in 3114 papers that were subsequently reviewed by the authors, of which 270 were considered eligible for the present work. From those publications, 29 were included in a more in-depth analysis according to criteria described below (Figure 2).

### Selection of Eligibility and Exclusion Criteria

The eligibility and exclusion criteria (Figure 2) were as follows: publication type (1); matrices studied (2); and extraction method using organic solvents (3). In line with the eligibility criteria selected, only journal articles with empirical data were considered (1); only studies reporting bioactivity assays using seaweeds were considered, and studies using seaweeds and mixed were also considered (2); and studies reporting assays with extracts obtained using organic solvents (e.g., n-hexane, diethyl ether, dichloromethane, n-butanol, chloroform, ethyl acetate, acetone, ethanol, and methanol) were considered (3). The following studies were excluded: reviews, book chapters, proceeding papers, conference papers, and notes (1); studies reporting bioactivity from organisms other than seaweeds (2); and studies using water extracts (3). A total of 270 publications were considered eligible, with these subsequently being screened using the following sub-criteria: only studies identifying an isolated complex lipid group, classes, or species, or reaching a molecular structure were considered for a more in-depth analysis to assess a structure–function relationship. After applying these sub criteria, 29 publications were selected, with these being discussed in detail in Section 3.1.

## 3. Results and Discussion

**Figure 3:**
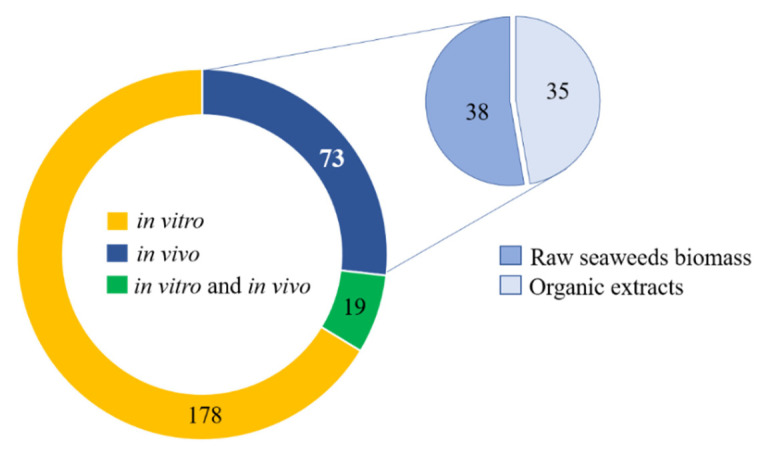
Number of eligible studies that recorded bioactivity on raw seaweed biomass or seaweeds organic extracts, distributed by type of performed assays (in vitro, in vivo and both in vitro and in vivo).

After applying the eligibility criteria adopted in the present work, 270 publications were considered for further analysis. These publications were evaluated taking in account the methodological approaches employed to perform bioassays, namely in vitro versus in vivo studies. Data analysis revealed that 178 publications referred to in vitro experiments, 73 to in vivo assays, and 19 included both in vitro and in vivo assays (Figure 3). It was also possible to record those in vivo assays included experimental work usually framed within two different approaches: (i) raw seaweed biomass; or (ii) organic extracts administrated intragastrical or in the diet as additives or feed supplements (Figure 3). Papers that described in vitro assays aimed to evaluate bioactive properties of organic extracts, and in some papers, complex lipids were identified or isolated. The papers that describe both in vitro and in vivo results, evaluated bioactive activities of organic extracts using in vitro assays and also the biological effects after oral administration performed mainly in animal models.

**Figure 4:**
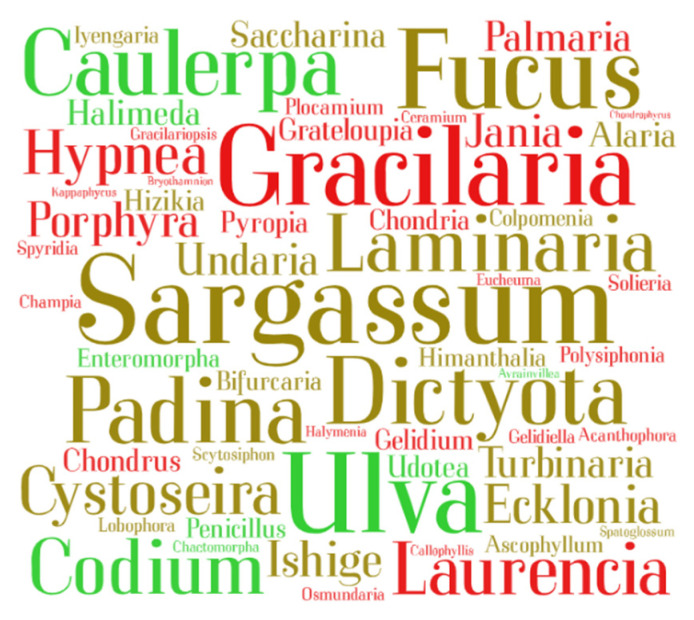
Word cloud assembled using the genera of seaweed species reported in the 270 eligible publications that reported bioactivity on raw seaweed biomass or seaweeds organic extracts. Genera featured with a larger size in the word cloud indicate that species within those genera were the ones mostly reported. Words in brown, green and red refer to genus within phylum Ochrophyta, Chlorophyta, and Rhodophyta, respectively (brown, green, and red seaweeds, respectively).

Data (270 publications) were plotted in a word cloud (Figure 4) featuring seaweed genus. This representation highlighted genera *Sargassum*, *Fucus*, *Dictyota*, and *Padina* (Ochrophyta; brown seaweeds), genera *Ulva* and *Codium* (Chlorophyta; green seaweeds), and genera *Gracilaria* (Rhodophyta; red seaweeds) as the most reported seaweeds with known bioactivities.

**Figure 5:**
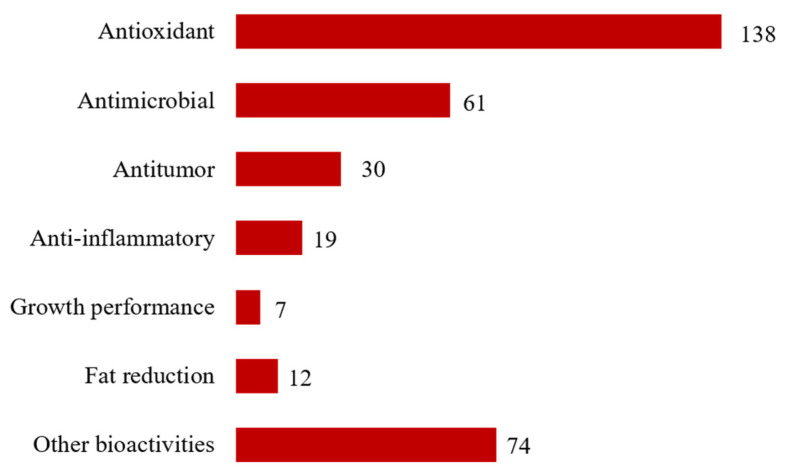
Number of eligible publications that reported bioactivity on raw seaweed biomass or seaweeds organic extracts.

To assess the biological effects reported in eligible studies, data was plotted considering the most frequently prospected bioactivities in the 270 eligible publications (Figure 5). Antioxidant activity (138 studies) was the most reported bioactivity, followed by antimicrobial (61 studies), antitumor (30 studies), anti-inflammatory (19 studies) activities, fat reduction (12 studies), and growth performance (7 studies). Other bioactivities included a wide range of different actions, which was not possible to group within a specific classification. It was also possible to record that most bioactivities reported were related to antioxidant or anti-inflammatory activities; the accurate bioactivity or bioactivities reported on each of these studies are summarized in Table S1.

Data (270 publications) was also ranked based on biomass of various seaweeds, or their extracts used in the bioassays performed, being grouped in five categories: studies using seaweed as raw seaweed biomass (category 1); studies using organic extracts (category 2); studies using organic extracts with identified complex lipids (category 3); studies of extracts enriched in isolated groups or classes of complex lipids (category 4); and studies of isolated complex lipid molecular species (category 5).

**Figure 6:**
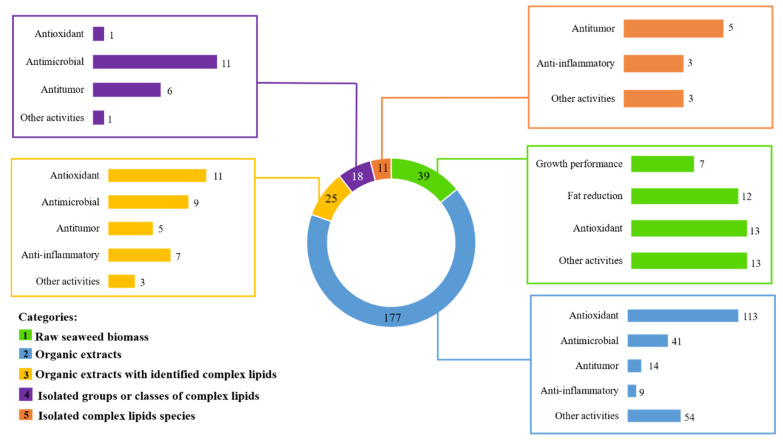
Ranking of eligible studies that reported bioactivity of raw seaweed or seaweeds organic extracts distributed by distinct categories.

In some of the selected categories (e.g., category 1 and 2) most studies did not highlight the identification of lipids, neither attributed the bioactivity reported to lipids. However, to our knowledge, the role of complex lipids in the observed bioactivity cannot be excluded. The distribution of eligible studies by category 1-5 and bioactivity assayed is summarized in Figure 6. Most studies were classified according to category 2 (177 studies), followed by category 1 (39 studies) and 3 (25 studies). Category 4 and 5 displayed a smaller number of studies (18 and 11, respectively). Category 1 included studies addressing the improvement of growth and/or immune system/health status, fat reduction, including reduction in hyperlipidemia/cholesterolemia/triglycerides, anti-obesity/anti-adipogenic effects; antioxidant and other activities (Table S1). Studies related with categories 2 to 5 pinpoint antioxidant, antitumor, anti-inflammatory, and antimicrobial (including antibacterial, antiviral, anti-protozoal, anti-microalgal, and anti-fouling) bioactivities. It is important to highlight that several studies reported more than one single bioactivity.

Antioxidant activity was most studied in categories 1 (13 studies out of 39), 2 (113 studies out of 177), and 3 (11 studies out of 25). In category 1, most studies that evaluated the antioxidant activity tested the inclusion of the raw seaweed biomass on diet, with no specification of the bioactive compound. In category 2, most studies tested organic extracts and were oriented towards phenolic compounds, which were recognized by their antioxidant properties. In category 3, the antioxidant activity was evaluated testing organic extracts with identified complex lipids, assigning the bioactivity to the whole extract and the synergic effect between molecules. The in vitro assays of antioxidant evaluation using free radical scavenging activities were one of the bioactivities more intensively investigated, likely because of well-established and easy-to-use methodologies. However, these *in chemico* assays have limited biological relevance considering the effect in the modulation of redox homeostasis of in vivo organisms. Therefore, additional studies are still needed using in vivo models, and measuring biologically relevant biomarkers of redox homeostasis, such as catalase, and superoxide dismutase enzymes, or addressing the proper value of seaweeds lipid antioxidant bioactivities.

Antimicrobial and antitumor activities were mostly studied on categories 4 (11 studies out of 18) and 5 (5 studies out of 11), respectively. Several studies reported the antimicrobial properties of lipid extracts from seaweeds. However, the majority of the studies reported only the estimation of inhibition of bacterial growth, lacking information on the identification of the bioactive lipids promoting such response and/or elucidating the mechanism of antimicrobial action. Interestingly, some studies reported antibacterial and antiviral activity of lipid extract from specific seaweeds and activities seem to be dependent on their origin. As society urgently needs new antibiotics to overcome the current scenario of antibiotic resistance, along with powerful new antiviral drugs to face future pandemics [7], it is urgent to further explore these bioactivities in seaweeds. Concerning antitumor activity, information is also scarce and lacks key information on putative structure function relationship.

**Figure 7:**
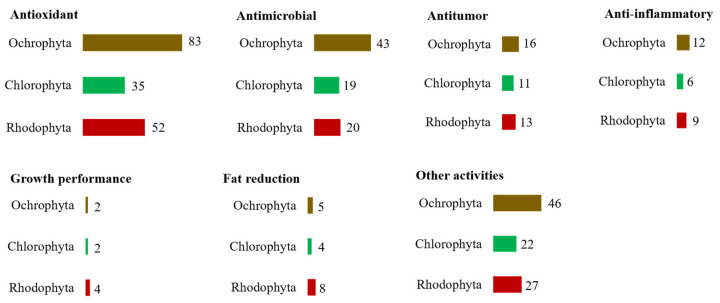
Number of eligible studies that reported bioactivity on raw seaweed biomass or seaweeds organic extracts distributed by bioactivities and seaweed phyla.

To unravel the most studied phyla of seaweeds, data (270 publications) were ranked considering how reported bioactivities were distributed over the phyla Ochrophyta, Chlorophyta, and Rhodophyta (Figure 7). Seaweed species belonging to the Ochrophyta were the most reported on antioxidant, antimicrobial, antitumor, and anti-inflammatory activities, followed by species within the Rhodophyta. Species within the Chlorophyta were the less studied.

**Figure 8:**
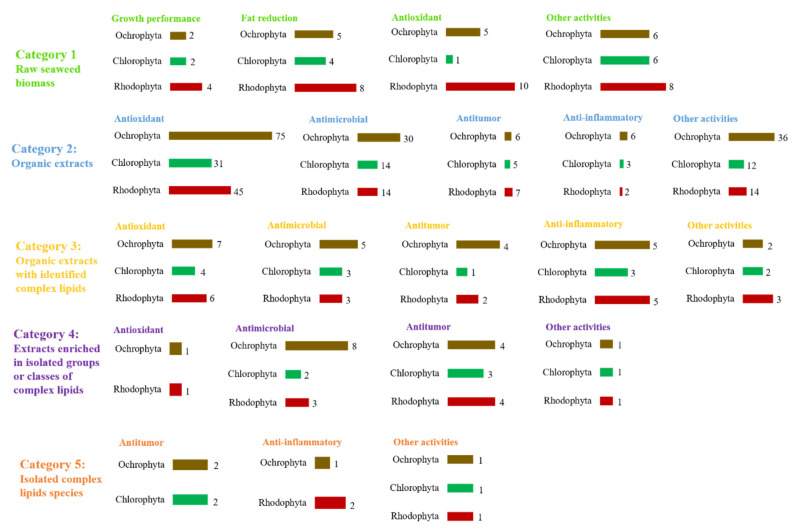
Number of eligible studies that reported bioactivity distributed over the five categories and seaweed phyla.

Bioactivity distributed by phylum combined with the five categories selected in the present study is plotted in Figure 8. Seaweeds within the Ochrophyta were the most screened to evaluate antioxidant, antimicrobial, antitumor, and anti-inflammatory bioactivities on category 2–4. On the other hand, seaweeds from the Rhodophyta were the most investigated to screen for growth performance, fat reduction, and antioxidant activity over criteria 1. Although with a lower number of studies on category 5, seaweed species within the Chlorophyta and Ochrophyta appeared as the most screened for antitumor activity. Seaweed species within the Rhodophyta were the most studied for anti-inflammatory activity under category 5.

In most studies (Table S1), the bioactivity reported for seaweed lipids was often associated with the most abundant molecules identified in organic extracts, or with other molecules detected by the methodology used for structural characterization (e.g., fatty acid identification by Gas Chromatography–Mass Spectrometry (GC-MS)). PUFA have been frequently identified as bioactive lipids in many studies because FA identification was the only approach used for extract characterization on those publications [31,43,44,45,46]. Nevertheless, this is an inadequate approach since FA commonly exist in low amounts as free FA and they are mostly esterified in complex lipids. Other studies tested extracts obtained with organic solvents, which also extract complex lipids. However, these studies only focused on the identification of well-known phytochemicals, which are present at a lower abundance in seaweeds, such as phenolic compounds, excluding the putative role of lipids and/or the synergic effect of other lipid-soluble compounds [47,48,49].

Knowledge progression of natural bioactive products and their application depends on the isolation of pure molecules to achieve a possible structure–function relationship [50,51]. While this is a very laborious and time-consuming task, it is also essential to understand specific biological effects of these biomolecules. Moreover, this task will also provide a new perspective to plan chemical synthesis and subsequent applications on different fields, such as in the pharmaceutical industry, aiming to add-value to seaweeds as natural sources of bioactive compounds. To date, few studies have tried to overcome this drawback. New studies being performed on bioassays using specific groups or class of seaweed lipids are scarce; although, they are paramount to isolate molecules to address a proper clarification of structure–bioactivity relationship. These studies are detailed bellow.

### 3.1. The Complex Lipids of Seaweeds as Derived Bioactive Phytochemicals

Studies addressing extracts enriched in isolated groups or classes of complexes lipids (category 4) and studies of isolated complex lipid species (category 5) are a minority. However, they provide a greater level of confidence concerning the bioactivity reported on complex lipids. These studies were selected for inclusion criteria following PRISMA-P workflow. Herein, they were ranked based on the bioactivities they evaluated.

#### 3.1.1. Antitumor Activity

Naturally occurring compounds have been tested for antiproliferative/cytotoxic, pro-apoptotic, anti-metastatic, and anti-neoplastic activities, among others [52,53,54].

Screening of antiproliferative activity is the most common approach to evaluate antitumor potential of complex lipids. Several cancer cell lines have been used including hepato [55,56], cervix [57], breast [56,58], leukemia [58,59], colon [58,60], lung [58,61], melanoma [62], and prostate and ovarian cancer [58]. The majority of these studies used lipid fractions enriched in a specific lipid group or class, obtained by using silica gel columns and solvents with different polarities. This approach was performed, for example, to evaluate the PLs fraction of the brown seaweed *Sargassum marginatum* inhibiting promyelocytic cells (HL-60) [59]. There is a huge variety of classes within PLs group that can contribute for bioactivity of these fractions; thus, the analysis of enriched lipid fractions solely provides a partial interpretation of results. Fractions enriched in GLs classes were isolated, allowing the identification of inhibitory activity against several cancer cells lines in digalactosyldiacylglycerol (DGDG) [60] and sulfoquinovosyldiacylglycero (SQDG) [55,56,57,60] enriched fractions (Table 1).

Few works have evaluated bioactivities of isolated lipid classes. The monogalactosyldiacylglycerol (MGDG) (MGDG 14:0_16:1) from the red seaweed *Solieria chordalis* and DGDG (14:0_18:3) from the green seaweed *Ulva armoricana* were found to have activity against NSCLC-N6 cancer cells [61]. However, to the best of our knowledge, the authors only identified the most abundant lipid species in the fraction, undervaluing other unidentified lipid species. Therefore, the antiproliferative activity of previous GLs molecular species could be incorrectly attributed.

**Figure 9:**
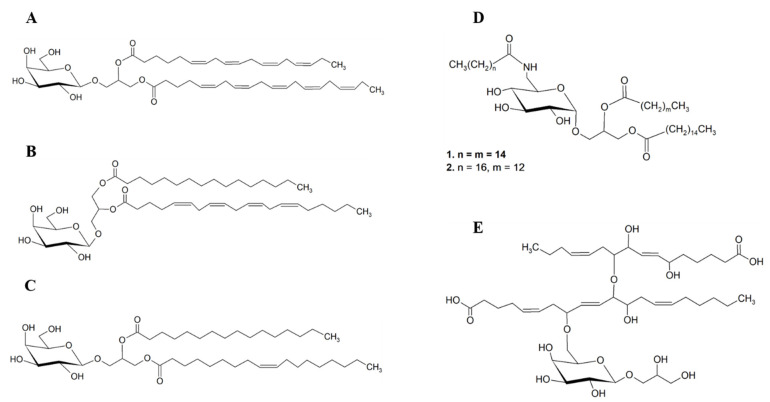
Chemical structures of bioactive complex lipids reported with antitumor activity. (**A**) 1-*O*-(5Z, 8Z, 11Z, 14Z, 17Z-eicosapentanoyl)-2-*O*-(6Z,9Z,12Z,15Z-octadecatetraenoyl)-3-*O*-*β*-d-galactopiranosyl-*sn*-glycerol MGDG (20:5/18:4) (brown seaweed *Fucus evanescence*); (**B**) 1-*O*-(palmitoyl)-2-*O*-(5Z, 8Z, 11Z, 14Z eicosatetraenoyl)-3-*O*-*β*-d-galactopyranosyl-glycerol MGDG (20:4/16:0) (red seaweed *Hydrolithon reinboldii*); (**C**) GDG (16:0, 18:1) (brown seaweed *Petalonia bingbamiae*) [63]; (**D**) *sn*-1,2-dipalmitoyl-3-(*N*-palmitoyl-6-deoxy-6-amino-*α*-d-glucosyl)-glycerol (1) and *sn*-1-palmitoyl-2-myristoyl-3-(*N*-stearyl-6-deoxy-6-aminoglucosyl)-glycerol (2); (**E**) Nigricanoside A (green seaweed *Avrainvillea nigricans*).

There are very few studies that achieved the isolation and identification of pure compounds, such as 1-*O*-(5Z, 8Z, 11Z, 14Z, 17Z-eicosapentanoyl)-2-*O*-(6Z,9Z,12Z,15Z-octadecatetraenoyl)-3-*O*-β-d-galactopiranosyl-*sn*-glycerol, (MGDG (20:5/18:4)) (Figure 9A) from the brown seaweed *Fucus evanescence* [62] with activity against malignant melanoma (SK-MEL-28), and 1-*O*-(palmitoyl)-2-*O*-(5Z, 8Z, 11Z, 14Z-eicosatetraenoyl)-3-*O*-β-d-galactopyranosylglycerol, (MGDG 20:4/16:0) (Figure 9B) from the red seaweed *Hydrolithon reinboldii*, which demonstrated inhibitory activity against a range of 12 cancer cell lines [58].

Along with the assessment of cell viability and the antiproliferative effect of lipid extracts, several biochemical approaches have also been developed in order to interrupt the cancer cells progression, including the inhibition of enzymes and disruption of mitotic process. The inhibition of DNA polymerases α was achieved by GLs species identified as galactosyldiacylglycerol esterified with the FAs C18:1 and C16:0 (GDG(18:1/16:0)) (Figure 9C) isolated from the brown seaweed *Petalonia bingbamiae* [63]. Likewise, the inhibition of MYT1 kinase by two GLs lipid species from unknown seaweed species were reported and these bioactive GLs species were identified as *sn*-1,2-dipalmitoyl-3-(*N*-palmitoyl-6-deoxy-6-amino-α-d-glucosyl)-glycerol and *sn*-1-palmitoyl-2-myristoyl-3-(*N*-stearyl-6-deoxy-6-aminoglucosyl)-glycerol (Figure 9D) [64]. The total synthesis of 1,2-dipalmitoyl-3-(*N*-palmitoyl-6′-amino-6′-deoxy-α-d-glucosyl)-*sn*-glycerol based on previous study [64], was achieved by Göllner and co-authors that confirmed those GLs lipids species as bioactive [65].

The species of GL isolated from the green seaweed *Avrainvillea nigricans*, named Nigricanoside A (Figure 9E), showed the capacity to arrest MCF-7 cells in mitosis, stimulating the polymerization of pure tubulin in vitro and thus inhibiting the proliferation of MCF-7 and HCT-116 cells [66]. The potent antimitotic activity of Nigricanoside A was seen without precedent among previously known GL.

**Table 1:** Lipid species extracted from seaweeds with antitumor activities. Extraction and characterization methodologies and cell lines used in in vitro assays are reported. Data is reported by phylum (Ochrophyta, Rhodophyta, Chlorophyta, or mixed phyla) and ranked by alphabetical order of seaweed species name within each phylum (or mixed phyla).

Study Category	Seaweed Species	Phylum	Lipid Species	Model and Obtained Results	Extraction Procedure	Identification/ Characterization	Ref.
Category 5	*Fucus* *evanescens*	Ochrophyta	MGDG (20:5/18:4)	Melanoma (SK-MEL-28), IC_50_ of 104 µΜ, (MTS assay)	Extraction solvents: ethanol (40 °C); deionized water, aqueous ethanol (70%), chloroform. Fractionation/isolation: silica gel, Sephadex LH-20 column chromatography	TLC; ESI-MS; ^1^H-, ^13^C-NMR; GC-FID, GC-MS	[62]
Category 5	*Petalonia* *binghamiae*	Ochrophyta	GDG (16:0/18:1)	Inhibition of DNA polymerase α, IC_50_ of 54 μM, (WST-1 assay)	Extraction solvents: acetone; ethyl acetate and water. Fractionation/isolation: silica gel, SephadexLH-20 column chromatography	GC-FID; EI mass; FABHR mass; ^1^H-, ^13^C- and DEPT- NMR	[63]
Category 4	*Sargassum horneri*	Ochrophyta	DGDD, SQDG,	Colon carcinoma (Caco-2), inhibition effect at 100 µΜ, (action improved with 1.0 mM NaBT)	Extraction solvents: chloroform, methanol, water (Bligh and Dyer). Fractionation: silica gel column chromatography	TLC, GC-FID	[60]
Category 4	*Sargassum marginatum*	Ochrophyta	PL	Human pro-myelocytic leukemia (HL-60), inhibition >70% at 40 µg mL^−1^ (trypan blue dye exclusion assay)	Extraction solvents: methanol, chloroform: methanol (1:1), water. Fractionation: silica gel column chromatography	GC-FID; GC-MS	[59]
Category 4	*Gracilaria corticata*	Rhodophyta	SQDG	Epithelioid cervix carcinoma (HeLa), IC_50_ of 106.88 μg mL^–1^ (MTT assay)	Extraction solvents: ethyl acetate/methanol (1:1), *n*-hexane, dichloromethane, butanol, water. Fractionation: silica gel column chromatography	GC-MS, TLC, ^1^H-^13^C-NMR (1H-1H COSY, DEPT, HSQC, HMBC spectra), HPLC	[57]
Category 5	*Hydrolithon reinboldii*	Rhodophyta	MGDG (20:4/16:0) (designated as Lithonoside)	Colon cancer (HCT116), prostate cancer (PC-3, LNcap-FGC, Du145), ovarian cancer (A2780/DDP-S), lung cancer (NCI-H446, SHP-77), leukemia (CCRF-CEM), breast cancer (BT-549, DU4475, MDA-MB-468, MDA-MB-231), average IC_50_ of 19.8 μM (MTS assay)	Extraction solvents: methanol, methanol: dichloromethane (1:1), methanol: water (9:1), hexane, ethyl acetate, butanol. Fractionation/isolation: semi-preparative reversed-phase HPLC, C18 HPLC	HPLC(C18)-Q-TOF-MS; ^1^H-, ^13^C-NMR (DEPT, COSY, HSQC, HMBC spectra)	[58]
Category 4	*Porphyra crispata*	Rhodophyta	SQDG	Liver carcinoma (HepG2), IC_50_ of 126 μg mL^−1^ (MTT assay)	Extraction solvents: ethanol. Fractionation: HP-20 column, DEAE-cellulose acetate column, TLC	GC-FID; TLC, normal-phase HPLC-ELSD	[55]
Category 5	*Avrainvillea nigricans*	Chlorophyta	Nigricanoside A	Breast adenocarcinoma (MCF-7) colon cancer (HCT-116) antimitotic activity, IC_50_ of 3 nM	Extraction solvents: methanol, water, ethyl acetate, hexane, dichloromethane. Fractionation/isolation: normal phase flash, Sephadex LH-20, reversed-phase flash column chromatographies, reversed-phase HPLC	HRESIMS; ^1^H-, ^13^C-NMR (DEPT, COSY, HSQC, HMBC spectra)	[66]
Category 4	*Solieria chordalis;* *Ulva armoricana*	Rhodophyta Chlorophyta	MGDG (14:0_16:1) DGDG (14:0_18:3)	Bronchopulmonary carcinoma (NSCLC-N6), IC_50_ of 23.5 μg mL^−1^ for MGDG (14:0_16:1) and IC50 of 24.0 μg mL^−1^ for DGDG (14:0_18:3) (MTT assay)	Extraction solvents: chloroform/methanol (1:1), water, dichloromethane, acetone, methanol. Fractionation: flash column chromatography	GC-MS; TLC; LC-MS	[61]
Category 4	*Dilophus fasciola;* *Galaxaura cylindrica;* *Laurencia popillosa;* *Taonia atomaria;* *Ulva fasciata,*	Rhodophyta Chlorophyta	Sulfolipid class	Hepato cellular carcinoma (Hep G2), IC_50_ in a range of 0.60 to 2.75 μg mL^–1^, Breast adenocarcinoma (MCF-7), IC_50_ in a range of 0.40 to 0.67 μg mL^–1^ (SRB assay)	Extraction solvents: methanol/chloroform (2:1). Fractionation: DEAE-cellulose column chromatography	IR; GC-FID; GC-MS; LC-MS/MS	[56]
Category 5	Unknown algal species		*sn*-1,2-dipalmitoyl-3-(*N*-palmitoyl-6-deoxy-6-amino-α-d-glucosyl)-glycerol; *sn*-1-palmitoyl-2-myristoyl-3-(*N*-stearyl-6-deoxy-6-aminoglucosyl)-glycerol	Inhibition of MYT1 kinase, IC_50_ of 0.12 and 0.43 μg mL^−1^	Extraction solvents: methanol, water, *n*-hexane, dichloromethane, butanol. Fractionation/isolation: Sephadex LH-20; RP-18 reverse phase silica gel;	^1^H-, ^13^C-NMR; MALDI-TOF-MS	[64]

#### 3.1.2. Anti-Inflammatory Activity

Inflammation is a multifactorial condition ubiquitously present in most diseases and particularly in non-communicable diseases. It involves a large number of identified mediators, comprising leukocyte cells that release specialized substances such as pro-inflammatory cytokines [67] and high levels of nitric oxide (NO) in response to the inflammatory process [68].

**Figure 10:**
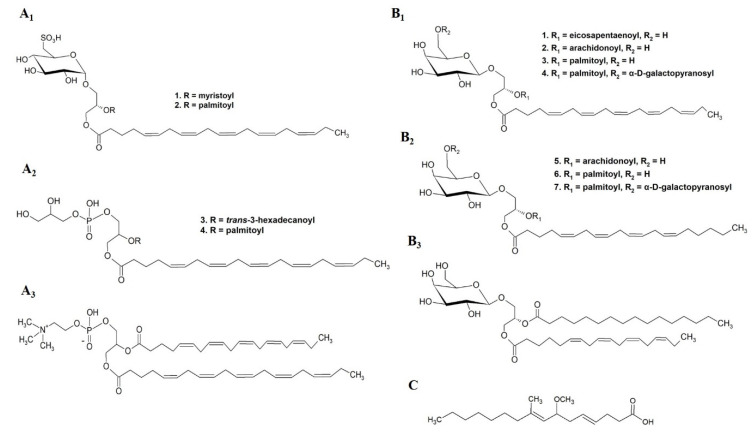
Chemical structures of bioactive complex lipids reported with anti-inflammatory activity: (**A1**) (2S)-1-*O*-eicosapentaenoyl-2-*O*-myristoyl-3-*O*-(6-sulfo-*α*-d-quinovopyranosyl)-glycerol SQDG (20:5/14:0) (1); (2S)-1-*O*-eicosapentaenoyl-2-*O*-palmitoyl-3-*O*-(6-sulfo-*α*-d-quinovopyranosyl)-glycerol SQDG(20:5/16:0) (2); (**A2**) 1-*O*-eicosapentaenoyl-2-*O*-*trans*-3-hexadecanoyl-3-phospho-(1′-glycerol)-glycerol PG(20:5/*trans*-16:1) (3); 1-*O*-eicosapentaenoyl-2-*O*-palmitoyl-3-phospho-(1′-glycerol)-glycerol PG(20:5/16:1) (4); (**A3**) 1,2-*bis*-*O*-eicosapentanoylglycero-3-phosphocholine PC(20:5/20:5) (red seaweed *Palmaria palmata*); (**B1**) (2S)-1,2-*bis*-*O*-eicosapentaenoyl-3-*O*-*β-*d-galactopyranosylglycerol MGDG(20:5/20:5) (1); (2S)-1-*O*-eicosapentaenoyl-2-*O*-arachidonoyl-3-*O-β-*d-galactopyranosylglycerol MGDG(20:5/20:4) (2); (2S)-1-*O*-eicosapentaenoyl-2-*O*-palmitoyl-3-*O-β-*d-galactopyranosylglycerol MGDG(20:5/16:0) (3); (2S)-1-O-eicosapentaenoyl-2*-O*-palmitoyl-3-*O*-(*β-*d-galactopyranosyl-6-1-*α*-d-galactopyranosyl)-glycerol DGDG (20:5/16:0)(4); (**B2**) (2S)-1,2*-bis*-*O*-arachidonoyl-3-*O-β-*d-galactopyranosylglycerol MGDG(20:4/20:4) (5); (2S)-1-*O*-arachidonoyl-2-*O*-palmitoyl-3-*O-β-*d-galactopyranosylglycerol MGDG(20:4/16:0) (6); (2S)-1-*O*-arachidonoyl-2-*O*-palmitoyl-3-*O*-(*β-*d-galactopyranosyl-6-1-*α*-d-galactopyranosyl)-glycerol DGDG(20:4/16:0) (7); (**B3**) (2S)-1-*O*-(6Z,9Z,12Z,15Z-octadecatetranoyl)-2-*O*-palmitoyl-3-*O*-*β-*d-galactopyranosylglycerol MGDG (18:4/16:0) (red seaweed *Chondrus crispus*); (**C**) 7-methoxy-9-methylhexadeca-4,8-dienoic acid (MMHDA) (brown seaweed *Ishige okamurae*).

NO is a potent pro-inflammatory mediator in over inflammation conditions [69]. On a small scale, and for research purposes, inhibition of NO, represents a protective effect of several anti-inflammatory compounds. The reduction in NO production from immune cells is assessed as a first step in the anti-inflammatory potential of natural products. Using this approach, several studies evaluated the anti-inflammatory activity of isolated and characterized seaweed lipid molecules (Table 2) including (2S)-1-*O*-eicosapentaenoyl-2-*O*-myristoyl-3-*O*-(6-sulfo-*α*-d-quinovopyranosyl)-glycerol SQDG (20:5/14:0), (2S)-1-*O*-eicosapentaenoyl-2-*O*-palmitoyl-3-*O*-(6-sulfo-*α*-d-quinovopyranosyl)-glycerol SQDG(20:5/16:0), 1-*O*-eicosapentaenoyl-2-*O*-*trans*-3-hexadecanoyl-3-phospho-(1′-glycerol)-glycerol PG(20:5/*trans*-16:1), 1-*O*-eicosapentaenoyl-2-*O*-palmitoyl-3-phospho-(1′-glycerol)-glycerol PG(20:5/16:1), and 1,2-*bis*-*O*-eicosapentanoylglycero-3-phosphocholine PC(20:5/20:5) (Figure 10(A1–A3)) from the red seaweed *Palmaria palmata* [70]; and isolated galactolipid species from the red seaweed *Chondrus crispus*, such as (2S)-1,2-*bis*-*O*-eicosapentaenoyl-3-*O*-*β*-d-galactopyranosylglycerol MGD(20:5/20:5), (2S)-1-*O*-eicosapentaenoyl-2-*O*-arachidonoyl-3-*O*-*β*-d-galactopyranosylglycerol MGDG(20:5/20:4), (2S)-1-*O*-eicosapentaenoyl-2-*O*-palmitoyl-3-*O*-*β*-d-galactopyranosylglycerol MGDG(20:5/16:0), (2S)-1-*O*-eicosapentaenoyl-2-*O*-palmitoyl-3-*O*-(*β*-d-galactopyranosyl-6-1-*α*-d-galactopyranosyl)-glycerol DGDG(20:5/16:0), (2S)-1,2-*bis*-*O*-arachidonoyl-3-*O*-*β*-d-galactopyranosylglycerol MGDG(20:4/20:4), (2S)-1-*O*-arachidonoyl-2-*O*-palmitoyl-3-*O*-*β*-d-galactopyranosylglycerol MGDG(20:4/16:0), (2S)-1-*O*-arachidonoyl-2-*O*-palmitoyl-3-*O*-(*β*-d-galactopyranosyl-6-1*α*-d-galactopyranosyl)-glycerol DGDG(20:4/16:0), and (2S)-1-*O*-(6Z,9Z,12Z,15Z-octadecatetranoyl)-2-*O*-palmitoyl-3-*O*-*β*-d-galactopyranosylglycerol MGDG(18:4/16:0) (Figure 10(B1–B3)) [71], which showed significant NO inhibition through down-regulation of inducible Nitric Oxide Synthase (iNOS). PUFA side chains, mainly EPA and arachidonic acid (AA), esterified to polar lipid structure seem to be relevant for their potent NO inhibition. Curiously, isolated PUFA, such as EPA, AA, and DHA, showed less NO inhibitory activity when compared to their esterified forms in polar lipid [70,71].

The capacity to inhibit phospholipase A2 (PLA2) has been linked to the efficacy for the treatment of inflammatory processes, since PLA2 hydrolyze membrane phospholipids releasing AA, the precursor of the pro-inflammatory mediators prostaglandins, thromboxanes, and leukotrienes [72,73]. Inhibition of PLA2 is the pharmacological mechanism of action of corticosteroids, a group of drugs with potent anti-inflammatory properties. The 7-methoxy-9-methylhexadeca-4,8-dienoic acid (MMHDA) (Figure 10C) isolated from the brown seaweed *Ishige okamurae* was tested in vitro for inhibition of PLA2 activity, and in vivo on edema and erythema induced in rat models. In both models, it demonstrated potent inhibitor of PLA2 activity and inflammation, with IC_50_ concentrations lower than the ones reported for rutin, a flavonoid model [74].

**Table 2:** Lipid species extracted from seaweeds with anti-inflammatory activities. Extraction and characterization methodologies and cell lines used in bioassays are reported. Data is reported by phylum (Ochrophyta, Rhodophyta, Chlorophyta, or mixed phyla) and ranked by alphabetical order of seaweed species name within each phylum (or mixed phyla).

Study Category	Seaweed Species	Phylum	Lipid Species	Model and Obtained Results	Compounds Extraction	Identification/Characterization	Ref.
Category 5	*Chondrus crispus*	Rhodophyta	MGDG(20:5/20:5) MGDG(20:5/20:4) MGDG(18:4/16:0) MGDG(20:5/16:0) MGDG(20:4/20:4) MGDG(20:4/16:0) DGDG(20:5/16:0) DGDG(20:4/16:0)	Raw 264.7 cells NO inhibition at 100 μM	Extraction solvents: methanol, water, ethyl acetate. Fractionation/isolation: SPE, HPLC (synergy MAX-RP column), semi-preparative HPLC	LC/MS; ^1^H-, ^13^C-NMR; GC; HRMS	[71]
	*Ishige okamurae*	Ochrophyta	MMHDA	*in vitro* inhibition of PLA2, IC_50_ of 1.9 μg mL−^1^ *in vivo* inhibition of oedema, IC_50_ of 3.5 mg mL−^1^ *in vivo* inhibition of erythema, IC_50_ of 4.6 mg mL−^1^	Extraction solvents: methanol, chloroform. Fractionation/isolation: Sephadex LH-20 column, silica gel column, reverse-phase HPLC, μBondapak C-18 column	HPLC (C18); GC-MS-QP5050A; EIMS;	[74]
	*Palmaria palmata*	Rhodophyta	SQDG(20:5/14:0) SQDG(20:5/16:0) PG(20:5/*trans*-16:1) PG(20:5/16:1) PC(20:5/20:5)	Raw 264.7 cells NO inhibition SQDG(20:5/14:0), IC_50_ of 36.5 µM SQDG(20:5/16:0), IC_50_ of 11.0 µM PG(20:5/*trans*-16:1), IC_50_ of 16.7 µM PG(20:5/16:0), IC_50_ of 42.9 µM PC(20:5/20:5), IC_50_ of 43.5 µM All species reduced iNOS expression >85% at 100 µM	Extraction solvents: methanol: chloroform (1:1), water, ethyl acetate. Fractionation/isolation: silica gel column chromatography; semi-preparative HPLC	ESI-MS; ^1^H-, ^13^C-NMR (COSY, HSQC HMBC spectra)	[70]

#### 3.1.3. Antimicrobial Activity

The emergence of antibiotic resistance of human pathogenic microorganisms and the need for new antiviral drugs has been a key driver for searching new antimicrobial compounds [75]. Complex lipids from seaweeds could play an active role in this field. In this section we describe the lipids from seaweeds with reported antibacterial, antiviral, anti-algal, anti-fouling, antifungal and anti-protozoal activities (Table 3). In spite of the range of antimicrobial activities tested, there is still opportunity to gain a more in-depth knowledge on this bioactive property of seaweed lipids, namely by testing against other strains of bacteria and virus that are major drivers of infection diseases

The GLs classes MGDG, DGDG, and SQDG from some species of *Laminaria* genus [76,77]; the brown seaweeds *Fucus evanescens* [78], *Alaria fistulosa* [76], *Saccharina cichorioides* [79]; and the red seaweed *Chondria armata* [80], demonstrated activity against a range of bacteria, yeast, and fungus. Likewise, sulfolipids classes from several seaweed species proved antibacterial activity [56]. In addition to antibacterial and antifungal activity, an isolated mixture of SQDG species from the brown seaweed *Lobophora variegata* showed anti-protozoal activity [81]. Isolated sub-fractions enriched in GL from the green seaweed *Ulva prolifera* [82] and the brown seaweed *Sargassum vulgare* [83] showed anti-algal and anti-fouling activities, respectively.

The studies surveyed pinpoint the evaluation of the complex lipid antiviral activity on Herpes simplex virus (HSV). The SQDG class from the red seaweed *Osmundaria obtusiloba*, the brown seaweed *Sargassum vulgare* and several species within genus *Laminaria* (brown seaweeds), were highlighted by its antiviral activity against HSV-1 [56,84,85] and HSV-2 [84,85]. The role of palmitic acid and sulfonate group on SQDG molecular structure was considered as relevant on activity against HSV virus and on cellular receptors [85].

Prospecting new antimicrobial compounds should follow a systemic protocol once the goal is to design solutions for human protection. Tested compounds must also show low toxicity against erythrocytes, which was evaluated in parallel in some studies that revealed hemolytic activity [76,77,78].

**Table 3:** Lipid species extracted from seaweeds with antimicrobial activities. Extraction and characterization methodologies and cell lines used in bioassays are reported. Data is reported by phylum (Ochrophyta, Rhodophyta, Chlorophyta, or mixed phyla) and ranked by alphabetical order of seaweed species name within each phylum (or mixed phyla).

Study Category	Seaweed Species	Phylum	Lipid Species	Activity (Microorganisms) and Obtained Results	Extraction	Identification/ Characterization	Ref.
Category 4	*Fucus evanescens*	Ochrophyta	MGDG, DGDG, SQDG classes	Antibacterial and antifungal (*Candida albicans, Fusarium* *oxysporum, Staphylococcus aureus, Escherichia coli*) Paper disk assay Unknown concentration	Extraction solvents: ethanol; ethanol:acetone (1:1); chloroform:ethanol (1:1); chloroform; water. Fractionation: silica gel column chromatography	TLC; GC-MS	[78]
	*Laminaria cichorioides*	Ochrophyta	MGDG, DGDG, SQDG classes	Antibacterial and antifungal (Safale S04, *Candida albicans, Fusarium oxysporum, Aspergillus niger, Staphyllococcus aureus, Escherichia coli*) Paper disk assay 3 mg mL^−1^	Extraction solvents: 96% ethanol, chloroform, water. Fractionation: silica gel column chromatography	TSC	[77]
	*Lobophora variegata*	Ochrophyta	Mixture of SQDG(16:0/14:0), SQDG16:0/16:0) and SQDG(16:0/18:1) species	Antiprotozoal (*Giardia intestinalis*, *Entamoeba histolytica* (Eh), *Trichomonas vaginalis*) Susceptibility assays IC_50_ of 3.9 μg mL^−1^ for *E. histolytica,* IC_50_ of 8.0 μg mL^−1^ for *T. vaginalis*, IC_50_ of 20.9 μg mL^−1^ for *G. intestinalis*	Extraction solvents: dichloromethane/methanol (7:3), methanol/water (9:1), hexane, chloroform, ethyl acetate and *n*-butanol. Fractionation: Sephadex LH20 column chromatograph	FAB-MS; ^1^H-*13*C-NMR (COSY, TOCSY, DEPT, HSQC and HMQC spectra)	[81]
	*Saccharina cichorioides*	Ochrophyta	Glycolipids (GL) group MGDG class	Antibacterial GL group: activity against *Staphylococcus aureus, Escherichia coli, Fusarium oxysporum,* and *Aspergillus niger* MGDG class: activity against *S. aureus* and *oxysporum* Paper disk assay Unknown concentration	Extraction solvents: ethanol: acetone (1:1, *v/v*), chloroform:ethanol (1:1, *v/v*). Fractionation: silica gel column	TLC	[79]
	*Sargassum vulgare*	Ochrophyta	Isolated SQDG fraction, identified SQDG(14:0/16:0), SQDG (16:0/16:0), SQDG (17:0/16:0), SQDG(18:1/ 16:0), SQDG(19:0/16:0), SQDG(23:0/17:0) species	Antiviral Inhibition HSV-1 and HSV-2 with maximum non-toxic concentrations (MNTC) of 50 μg mL−^1^ and viral inhibition index (VII) in a range of 96 (minimum) to 99.9% (maximum) Titer reduction assay	Extraction solvents: chloroform/methanol (2:1 and 1:2). Fractionation: silica column chromatography.	TLC; ESI-MS; ^1^H-, ^13^C-NMR	[85]
	*Sargassum vulgare*	Ochrophyta	Fraction enriched in MGDG (16:0/19:1) DGDG (16:0/16:1) SQDG (16:0/19:0) species	Antifouling Biofilm-forming marine bacteria (*Pseudoalteromonas elyakovii, Halomonas marina, Shewanella putrefaciens* and *Polaribacter irgensii*) and marine microalgae (*Chlorarachnion reptans*, *Pleurochrysis roscoffensis*, *Exanthemachrysis gayraliae, Cylindrotheca closterium*, and *Navicula jeffreyi*) MIC in a range of 0.01 to >10 μg/mL	Extraction solvents: chloroform/methanol (2:1 and 1:2), water. Fractionation: silica gel column chromatography.	HPTLC silica gel; TLC; LC-MS	[83]
	*Alaria fistulosa, Laminaria bongardiana,* *Laminaria longipes,* *Laminaria yezoensis*	Ochrophyta	MGDG, DGDG, SQDG classes	Antibacterial (*Staphylococcus aureus, Escherichia coli*) and antifungal (*Candida albicans, Fusarium oxysporum*), Paper disk assay Unknown concentration	Extraction solvents: ethanol; ethanol:acetone (1:1); chloroform:ethanol (1:1); chloroform; water. Fractionation: silica gel column chromatography.	TLC; GC-MS	[76]
	*Chondria armata*	Rhodophyta	Glycolipids (GL) group, identified the species 1-oleoyl-2-palmitoyl-3-*O*-(linolenyl-6′-galactosyl)-glycerol; 2-*O*-palmitoyl-3-*O*-(6′-sulfoquinovopyranosyl)-glycerol and 3-digalactosyl-2-palmitoyl glycerol	Antibacterial (*Klebsiella* sp., *Shigella flexineri*, *Vibrio cholerae*) and antifungal (*Candida albicans, Cryptococcus neoformans, Aspergillus fumigatus*) Paper disk assay impregnated with extract in a range of 65–130 μg/disk	Extraction solvents: methanol, chloroform, n-butanol and water. Fractionation: Sephadex LH20 for gel filtration, silica gel column chromatography, RP-18 column	TLC; QSTARXL MS/MS; ^1^H-, ^13^C-NMR (COSY, HMQC, and HMBC spectra)	[80]
	*Osmundaria* *obtusiloba*	Rhodophyta	SQDG class	Antiviral Inhibition HSV-1 and HSV-2 with 50% of effective concentration (EC_50_) values of 42 μg mL^−1^ to HSV-1 and 12 μg mL^−1^ to HSV-2 Titer reduction assay	Extraction solvents: acetone, chloroform/methanol (2:1 and 1:2). Fractionation: silica gel column chromatography, preparative TLC.	TLC; ESI-MS; ^1^H-, ^13^C-NMR (HSQC, COSY and TOCSY spectra)	[84]
	*Ulva prolifera*	Chlorophyta	Enriched subfraction on MGMG(18:0) MGMG(16:0) MGDG(16:0/18:1)	Antialgal: Inhibition > 50% of red tide microalgae (*Karenia mikimitoi, Skeletonema costatum, Alexandrium tamarense, Heterosigma akashiwo, Prorocentrum donghaiense*) at concentration of 28.8 μg mL^−1^	Extraction solvents: methanol, water, ethyl acetate Fractionation: silica gel column, Sephadex LH-20, preparative TLC.	TLC	[82]
	*Dilophys fasciola, Galaxoura cylindriea,* *Laurencia popillosa,* *Taonia atomaria,* *Ulva fasciata*	Ochrophyta Rhodophyta Chlorophyta	Sulfolipids classes	Antiviral: Inhibition HSV-1, IC_50_ ranged from 15 to 25 μg mL^–1^ (plaque reduction assay) Antibacterial (*Bacillus subtilis, Escherichia coli*) with MIC in a range of 40 to 80 μg mL^–1^ for *G. cylindriea*, *U. fasciata,* and *T. atomaria* (agar diffusion assay)	Extraction solvents: methanol:chloroform (2:1, *v/v*) Fractionation: DEAE-cellulose column chromatography.	IR, GC MS/MS, LC-MS/MS.	[56]

#### 3.1.4. Other Bioactivities Attribute to Seaweed Lipids

Complex lipids from seaweeds have showed a broad spectrum of bioactivates (Table 4), including antioxidant activity associated with GL and PL groups from the red seaweed *Solieria chordalis* and the brown seaweed *Sargassum muticum*, and evidenced through in vitro free radical scavenging activity [86]. However, the study did not characterize the compounds in the isolated fractions, which raises doubts about their purity and possible interference of other compounds.

Fractionated lipid classes, such as MGDG, were suggested to play an important role in the design of optimized nanoparticulate tubular immune-stimulating complexes. Sanina et al. (2021) found different degrees of effectiveness on anti-porin response, porin conformation, and cytokine profile of MGDG from different phyla with different FA composition [87].

**Figure 11:**
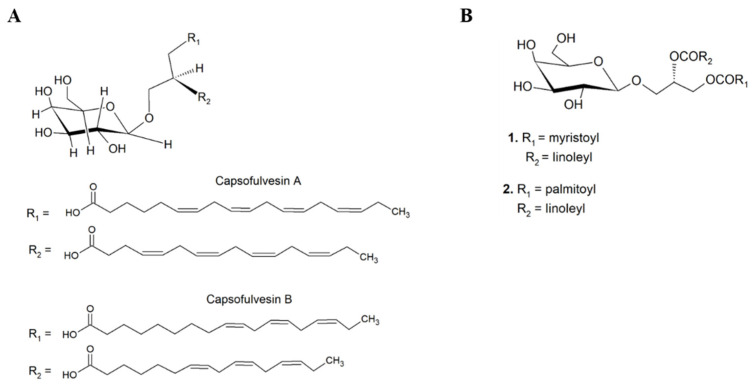
Chemical structures of bioactive complex lipids reported with anti-diabetic and anti-obesity activities. (**A**) (2S)-l-*O*-(6Z,9Z,12Z,15Z-octadecatetraenoyl)-2-*O*-(4Z,10Z,13Z-hexadecatetraenoyl)-3-*O*-β-d-galactopyranosylglycerol and (2S)-l-*O*-(9Z,12Z,15Z-octadecatrienoyl)-2-*O*-(10Z,13Z-hexadecadienoyl)-3-*O*-β-d-galactopyranosylglycerol capsofulvesin A and B (green seaweed *Capsosiphon fulvescens*); (**B**) (2S)-1-*O*-myristoyl-2-*O*-linoleyl-3-*O*-β-d-galactopyranosyl-*sn*-glycerol MGDG(14:0/18:2) (1) and (2S)-1-*O*-palmitoyl-2-*O*-linoleyl-3-*O*-β-d-galactopyranosyl-*sn*-glycerol MGDG(16:0/18:2) (2) (brown seaweed *Sargassum horneri*).

A study that bio-prospected and isolated bioactive molecular species from the green seaweed *Capsosiphon fulvescens* highlighted two GL species: (2S)-l-*O*-(6Z,9Z,12Z,15Z-octadecatetraenoyl)-2-*O*-(4Z,10Z,13Z-hexadecatetraenoyl)-3-O-*β-*d-galactopyranosylglycerol and (2S)-l-*O*-(9Z,12Z,15Z-octadecatrienoyl)-2-*O*-(10Z,13Z-hexadecadienoyl)-3-*O*-*β-*d-galactopyranosylglycerol (designated by capsofulvesin A and B, respectively) (Figure 11A) that showed capacity to inhibit rat lens aldose reductase (RLAR), thus showing potential for application as anti-diabetic agents [88]. The inhibitory effect on lipid accumulation of (2S)-1-*O*-myristoyl-2-*O*-linoleyl-3-*O*-*β*-d-galactopyranosyl-*sn*-glycerol MGDG (14:0/18:2) and (2S)-1-*O*-palmitoyl-2-*O*-linoleyl-3-*O*-*β*-d-galactopyranosyl-*sn*-glycerol MGDG (16:0/18:2) glycolipids species (Figure 11B) from the brown seaweed *Sargassum horneri* was also reported in 3T3-L1 adipocytes [89]. These two MGDG species have in common the presence of linoleic acid (LA) (18:2 *n*-6) on *sn*-2 FA chain position, and when compared to other isolated MGDG species they were the most effective. Thus, this study suggested that LA on the *sn-2* position of MGDG species played an important role on the inhibition of triglyceride accumulation in this biological model.

A human sperm motility stimulating activity was achieved by an isolated sulfono-glycolipid (named by S-ACT-1) from the red seaweed *Gelidiella acerosa*, whose molecular structure was not evidenced [90]

**Table 4:** Lipid species extracted from seaweeds with other activities. Extraction and characterization methodologies and tested bioactivities are reported. Data is reported by phylum (Ochrophyta, Rhodophyta, Chlorophyta, or mixed phyla) and ranked by alphabetical order of seaweed species name within each phylum (or mixed phyla).

Study Category	Seaweed Species	Phylum	Lipid Species	Activity and Action	Extraction	Identification/Characterization	Ref.
Category 4	*Solieria chordalis*, *Sargassum muticum*	Rhodophyta Ochrophyta	Glycolipids (GLs) and Phospholipids (PLs) groups	Antioxidant through DPPH free radical scavenging activity *Solieria chordalis*: GL with EC_50_ in a range of 0.9 to >5 mg mL^−1^ PL with EC_50_ in a range of 1.1 to >5 mg mL^−1^ *Sargassum muticum* GL with EC_50_ in a range of 0.9 to 4.1 mg mL^−1^ PL with EC_50_ in a range of 1 to 4.8 mg mL^−1^	Extraction solvents: chloroform/methanol (1/1) or supercritical carbon dioxide pure or with 2% or 8% of ethanol. Fractionation: silica gel column chromatography.	No characterization	[86]
	*Ahnfeltia tobuchiensis*, *Laminaria japonica, Sargassum pallidum*, *Ulva fenestrata*	Rhodophyta Ochrophyta Chlorophyta	MGDG class	Regulation of the immunogenicity of protein antigen in the content of TI-complexes	Extraction solvents: chloroform, methanol. Fractionation: silica gel column chromatography, purified by preparative silica TLC.	GC-FID	[87]
Category 5	*Capsosiphon* *fulvescens*	Chlorophyta	Capsofulvesin A and B	Anti-diabetic Rat lens aldose reductase (RLAR) inhibitory assay capsofulvesin A: IC_50_ of 52.53 μM capsofulvesin B: IC_50_ of 101.92 μM	Extraction solvents: 95% ethanol at 80 °C, water, partitioned dichloromethane, ethyl acetate, and n-butanol. Fractionation/isolation: silica gel column chromatography, reversed-phase (RP-C18) chromatography.	^1^H-, ^13^C-NMR	[88]
	*Sargassum horneri*	Ochrophyta	MGDG(14:0/18:2) MGDG(16:0/18:2)	Inhibitory effects on triglyceride and free fatty acids accumulation in 3T3-L1 adipocytes at concentration of 10 μM	Extraction: 70% alcohol, ethyl acetate. Fractionation/isolation: vacuum liquid chromatography (VLC) over silica gel, Sephadex LH-20, flash silica gel column chromatography	TLC; ^1^H-, ^13^C-NMR; GC-FID; HPLC−MS/MS	[89]
	*Gelidiella acerosa*	Rhodophyta	SQDG (S-ACT-1)	Human sperm motility stimulating activity	Extraction: dichloromethane: methanol (1:1). Fractionation/isolation: Sephadex LH-20	TLC; ^1^H-, ^13^C-NMR, IR	[90]

## 4. Concluding Remarks and Future Prospects

Seaweeds remain largely untapped reservoirs of natural bioactive molecules [10]. In fact, more than 11,300 species of seaweeds are reported on Algabase, of which only 42 species were surveyed on category 4 (studies of extracts enriched in isolated groups or classes of complex lipids) and category 5 (studies of isolated complex lipid molecular species), most of them within the Ochrophyta phylum. This reveals that the bioprospecting potential of seaweed lipids remains largely untapped.

Complex lipids from seaweeds are emerging as bioactive molecules with hidden potential; however, their exploitation is far from being optimized and their action mechanisms are still poorly understood. This figure is likely to change as more seaweeds have their bioactive complex lipids characterized and more mechanism-oriented studies are performed.

To date, not only do most studies lack a systematic research approach, but most of the lipid bioactivities already identified refer to total lipid extracts. Indeed, only a few studies have achieved molecular isolation and characterization of bioactive lipids. Interestingly, complex lipids isolated from seaweed species with reported bioactivity have been classified mainly as GLs species. This systematic analysis pinpoints the promising results of naturally occurring GLs in seaweeds, with emphasis to their antitumor and anti-inflammatory potential. The advances of emerging food/feed, nutraceutical, cosmeceutical, pharmaceutical, and complementary medicine research fields [91,92,93], as well as biological and experimental sciences, will contribute to boost structural characterization of complex lipids and to link lipid structure and bioactivity through different mechanisms of action.

Regardless of their polyphyletic nature, it is unquestionable that seaweeds as a whole, remain an important reservoir of lipid phytochemicals. Despite the low abundance of these biomolecules in seaweeds, they remain largely uncharacterized and unexplored. Complex lipids from seaweeds offer an unmatched chemical diversity and structural complexity when compared to terrestrial phytochemicals. It seems that seaweeds species or genera feature unique lipidomes, which likely enhances the potential number of target applications. Lipidomic characterization strategies using high-resolution apparatus, such as mass spectrometry, can be paramount to unleash the true potential of these biomolecules. The species-specific lipidome for each seaweed could be applied to the production of target bioactive lipids. Otherwise, isolated bioactive complex lipids can be used as a large-scale synthesis model. While some of their natural chemotherapy diversity has already been studied, resulting in open access and proprietary compound libraries, there is still a multitude of lipids from algal origin that have hardly been characterized. The potential of these biomolecules to develop new products and processes is certainly far from being exhausted. It is expected that the bioprospecting of seaweed extracts enriched in active lipids for the formulation of high-end products can foster the added value of seaweed biomass production.

Under this scope it will be possible to put forward innovative processes for the production of farmed seaweeds biomass under controlled conditions, as these will allow to target new markets and consumers under a circular and sustainable blue bioeconomy framework.

## Supplementary Materials

The following are available online at https://www.mdpi.com/article/10.3390/md19120686/s1, Table S1: Eligible studies distributed by title, published year, doi (when applicable), seaweeds species, genus, phylum, bioactivity reported, and category where they were inserted.
